# *Trichinella britovi* muscle larvae and adult worms: stage-specific and common antigens detected by two-dimensional gel electrophoresis-based immunoblotting

**DOI:** 10.1186/s13071-018-3177-x

**Published:** 2018-11-12

**Authors:** Sylwia Grzelak, Bożena Moskwa, Justyna Bień

**Affiliations:** Witold Stefański Institute of Parasitology, Polish Academy of Sciences, Twarda 51/55, 00-818 Warsaw, Poland

**Keywords:** *Trichinella britovi*, Adult worm, Muscle larvae, 2-DE, Mass spectrometry, Immunoblotting

## Abstract

**Background:**

*Trichinella britovi* is the second most common species of *Trichinella* that may affect human health. As an early diagnosis of trichinellosis is crucial for effective treatment, it is important to identify sensitive, specific and common antigens of adult *T. britovi* worms and muscle larvae. The present study was undertaken to uncover the stage-specific and common proteins of *T. britovi* that may be used in specific diagnostics.

**Methods:**

Somatic extracts obtained from two developmental stages, muscle larvae (ML) and adult worms (Ad), were separated using two-dimensional gel electrophoresis (2-DE) coupled with immunoblot analysis. The positively-visualized protein spots specific for each stage were identified through liquid chromatography-tandem mass spectrometry (LC-LC/MS).

**Results:**

A total of 272 spots were detected in the proteome of *T. britovi* adult worms (Ad) and 261 in the muscle larvae (ML). The somatic extracts from Ad and ML were specifically recognized by *T. britovi*-infected swine sera at 10 days post infection (dpi) and 60 dpi, with a total of 70 prominent protein spots. According to immunoblotting patterns and LC-MS/MS results, the immunogenic spots recognized by different pig *T. britovi*-infected sera were divided into three groups for the two developmental stages: adult stage-specific proteins, muscle larvae stage-specific proteins, and proteins common to both stages. Forty-five Ad proteins (29 Ad-specific and 16 common) and thirteen ML proteins (nine ML-specific and four common) cross-reacted with sera at 10 dpi. Many of the proteins identified in Ad (myosin-4, myosin light chain kinase, paramyosin, intermediate filament protein B, actin-depolymerizing factor 1 and calreticulin) are involved in structural and motor activity. Among the most abundant proteins identified in ML were 14-3-3 protein zeta, actin-5C, ATP synthase subunit d, deoxyribonuclease-2-alpha, poly-cysteine and histide-tailed protein, enolase, V-type proton ATPase catalytic and serine protease 30. Heat-shock protein, intermediate filament protein ifa-1 and intermediate filament protein B were identified in both proteomes.

**Conclusions:**

To our knowledge, this study represents the first immunoproteomic identification of the antigenic proteins of adult worms and muscle larvae of *T. britovi*. Our results provide a valuable basis for the development of diagnostic methods. The identification of common components for the two developmental stages of *T. britovi* may be useful in the preparation of parasitic antigens in recombinant forms for diagnostic use.

## Background

Trichinellosis is an important food-borne parasitic worldwide zoonosis caused by nematodes belonging to the genus *Trichinella* and is known to have high socioeconomic and medical significance. Humans typically acquire trichinellosis through the consumption of raw or improperly-processed meat of either farmed or wild animals containing infective muscle larvae (ML) of *Trichinella* [1–3]. The entire life-cycle of the parasite takes place in a single host. *Trichinella* displays three major antigenic stages: muscle larvae (ML), adult worms (Ad), and newborn larvae (NBL). Muscle larvae ingested with animal-derived meat are released into the host stomach upon the activation of digestive enzymes; they then migrate to the epithelial cells of the small intestine where they molt and transform into adult worms (Ad) within 48 hours post-infection (pi). Newborn larvae (NBL) are released after five days post-infection (dpi) and move through the lymphatic vessels to reach the striated muscle, where they grow and develop into encapsulated and non-encapsulated forms [4, 5]. All developmental stages of *Trichinella* elicit a protective immune response, as well as antigens which can be used for serological detection of *Trichinella* spp. infection. Several reports note that the *Trichinella* antigens produced by adult worms, new-born larvae and muscle larvae are stage-specific [6–8]. Our previous study indicated that together with stage-specific proteins, *T. spiralis* produces species-specific and common proteins for each developmental stage [9–11]. Although a few *Trichinella* antigens have been fully characterized, the complex interactions between the parasite and the host’s immune system are not yet fully understood [12–16]. Thus, there is still a need to find other parasite proteins which may play an important role during the establishment of infection, which influence immune evasion strategies or modulate the host response. Recent studies have shown that a serine protease inhibitor released by *T. spiralis* may allow it to escape immune attack, and is related to the survival and colonization of the parasite in the hosts [17]. Identification of these proteins is not only important for understanding parasite-host interrelations, but is also a key factor in the development of serological diagnostic methods for species-specific differentiation and for detecting early-stage infection.

The combination of two-dimensional gel electrophoresis (2-DE) and mass spectrometry has been widely used to characterize the protein profiles of various *Trichinella* species [9, 18–21]. When used together with immunoblotting, the techniques enable the identification of the proteins that induce immune response and which could be used for immunodiagnosis. This immunoproteomics tool has previously been used to determine both the characteristics of immunogenic proteins and the serological response directed against parasites, such as *Schistosoma japonicum* [22], *Toxoplasma gondii* [23], *Ascaris lumbricoides* [24] and *Taenia solium* [25]. As *T. spiralis* is considered the main etiological agent of most human infections and deaths, most studies have focused only on the identification of potentially immunogenic proteins expressed by *T. spiralis* stages [20, 26–29]. Although *T. spiralis* is commonly used as a representative species of the genus *Trichinella*, *T. pseudospiralis*, *T. nativa* and the T8 genotype, have also been described as being valuable sources of information regarding the parasite proteins needed for the development of immunological diagnostics [18, 19, 30].

Over the years, numerous cases with trichinellosis have been attributed to *T. britovi*, considered the second-most common species of *Trichinella and one* that may affect human health [31–36]. Although the clinical and biological features observed during human infection caused by *T. spiralis* and *T. britovi* are different, it is not possible to attribute these features to a single species because the number of infective larvae is unknown. *Trichinella spiralis* infections are typically more severe than those caused by *T. britovi*, and the main distinctions between the two types of infections were that patients infected with *T. spiralis* displayed a longer duration of parasite-specific IgG, increased CPK levels, and a more severe intestinal symptomatology than those infected with *T. britovi*. This could be due to the fact that the fecundity of *T. britovi* females is lower than those of *T. spiralis* [36]. Our previous proteomic study of the excretory-secretory proteins of *T. britovi* muscle larvae found that the 5'-nucleotidase and serine protease may be potential proteins for diagnosis [9]. Currently, little is known about the protein profile shared by all developmental stages of *T. britovi*. Therefore, there is a need for more information about common and stage-specific *T. britovi* proteins to aid the development of species-specific diagnostics, and to better understand the adaptation of *T. britovi* to a parasitic niche and its host-parasite relationship.

The aim of the present study was to identify the *T. britovi* proteins that may be used in specific diagnostics. Somatic antigen extracts obtained from two developmental stages of *T. britovi*, muscle larvae (ML) and adult worms (Ad), were separated by two-dimensional gel electrophoresis (2-DE) coupled with immunoblot analysis. In addition, any positively-visualized proteins specific for each stage were further identified by liquid chromatography-tandem mass spectrometry (LC-LC/MS).

## Methods

### Experimental animals and collection of *T. britovi* adult worms and muscle larvae

The *T. britovi* nematodes had been maintained by several passages in male C3H mice at the Institute of Parasitology, PAS. To generate ML and Ad forms of *T. britovi*, the mice were orally infected with a dose of 700 ML *T. britovi*. ML were collected 42 days post-infection (dpi), and Ad were collected at 4 dpi. Muscle larvae of *T. britovi* were recovered by HCl-pepsin digestion from the previously-infected mice [37]. The recovered ML were subsequently purified several times with water through succeeding steps of sedimentation in cylinders. After the final sedimentation, the ML were collected into 1.5 ml tubes. The larval pellet was extensively washed three times in phosphate-buffered saline (PBS) supplemented with antibiotics (50 U/ml penicillin, 50 μg/ml streptomycin). The adult worms were collected from the small intestine of C3H mice (3–4 months-old). Briefly, after recovery, the intestines were washed with sterile water with the use of a syringe, cut longitudinally and crosswise into 1–2 cm pieces, placed on a mesh in a conical dish filled with RPMI 1640 medium (Sigma-Aldrich Chemie GmbH, Steinheim, Germany) supplemented with 25 mM HEPES, 2 mM L-glutamine, antibiotics (50 U/ml penicillin, 50 μg/ml streptomycin) and incubated for three hours at 37 °C. Any Ad worms located on the bottom of the dish were then collected into 15 ml tubes, and washed three times with PBS supplemented with antibiotics. The *T. britovi* stages were then stored at -70 °C before protein extraction and proteomic analysis.

### Protein extraction

The same protein sample preparation procedure was used for both *T. britovi* stages. After thawing, the collected *T. britovi* ML and Ad were again extensively washed three times in PBS and then suspended in a lysis buffer (8 M Urea, 4% CHAPS, 40 mM Trizma base), supplemented with protease inhibitor cocktail (Roche, Berlin, Germany). The protein extract was then homogenized in glass Potter-homogenizer and disintegrated by sonication three times for 10 s. The lysis extract was clarified by centrifugation at 14,000× *g* at 4 °C for 15 min. The supernatant was collected, placed in new 1.5 ml tubes, and protein concentration was measured with the use of a NanoDrop-1000 Uv/Vis Spectrometer (NanoDrop Technologies, Wilmington, USA). The proteins were frozen at -70 °C for further analysis.

### Two dimensional gel electrophoresis (2-DE)

Three replicates of *T. britovi* protein samples were run in parallel on three immobilized pH-gradient IPG strips (RioRad, Hercules, USA). The 100 μg samples of previously prepared protein extracts from *T. britovi* Ad and ML were purified with the 2-D Clean-Up Kit (GE Healthcare, New Jersay ,USA) in accordance with the manufacturer’s protocol. After the final centrifugation step, the protein pellets were rehydrated overnight in 250 μl of 2-D Starter Kit Rehydration/Sample Buffer (BioRad, Hercules, USA) and loaded onto a 7 cm pH 3-10 IPG strips (BioRad, Hercules, USA) for first dimension separation. The protein samples were separated in accordance with their pI values through isoelectric focusing (IEF) using a Protean IEF Cell (BioRad) device at 20 °C as follows: first step 15 min at 250 V; second step rapid ramping to 4000 V for two hours; and third step for 15,500 Vhrs (current limit of 50 μA/IPG strip). After focusing, the strips were submitted for two steps of equilibration, the first for 25 min in ReadyPrep 2-D starter Kit Equilibration Buffer I, containing DTT (BioRad, USA), and the second for 25 min in ReadyPrep 2-D Starter Kit Equilibration Buffer II containing iodoacetamide (BioRad, USA) instead of DTT. The two-dimensional SDS-PAGE was run using 12% acrylamide separating gels and 4% polyacrylamide stacking gels in a Mini-PROTEAN Tetra Cell electrophoresis chamber (BioRad, USA) at 200 V for approximately 50 min. The PageRuler Unstained Protein Ladder (Thermo Fisher Scientific, Massachusetts, USA) was loaded onto each gel as a weight marker. All gels were separated in the same conditions.

### Silver staining and 2-DE immunoblotting

After 2-DE electrophoresis gels were silver-stained using PlusOne Silver Staining Kit (GE Healthcare) in accordance with manufacturer’s protocol, while those used for 2-DE immunoblotting were not stained. The obtained gels were scanned with ChemiDoc MP system (BioRad, USA) and analyzed in Image Lab 5.2.1. software (BioRad, USA).

In addition, proteins from unstained gels were transferred onto Immuno-Blot polyvinylidene fluoride (PVDF) membranes (BioRad) by a wet transfer system (BioRad, USA) at 95 V for one hour in cool conditions. The PVDF membranes with the Ad and ML proteins were blocked in Pierce Protein-Free T20 (TBS) Blocking Buffer (Thermo Fisher Scientific) for one hour at room temperature. Following this, the PVDF membranes were incubated overnight at 4 °C with *T. britovi*-infected pig sera (dose of 20,000 ML) diluted 1:100, at 10 dpi and 60 dpi. Adult worm proteins transferred onto the membrane were treated with antisera taken at 10 dpi while the ML proteins were treated with antisera from 10 dpi and 60 dpi. The secondary antibody HRP-conjugated goat anti-pig IgG were diluted 1:35 000 (Sigma-Aldrich, Louis, USA). The uninfected sera were used as parallel negative controls. The negative control experiment used the same method as mentioned above. The immunoreactive proteins were visualized on a film using a Super Signal West Pico Chemiluminescent Substrate (Thermo Fisher Scientific, Walthman, USA) according to the provided instruction. Reproducibility of the immune recognition was verified by repeating the immunoblot at least three times.

### LC-MS/MS

Spots of interest visible on the films were gently excised from compatible silver-stained gels and analyzed by liquid chromatography coupled to a mass spectrometer in the Laboratory of Mass Spectrometry, Institute of Biochemistry and Biophysics, Polish Academy of Sciences (Warsaw, Poland). Samples were concentrated and desalted on a RP-C18 pre-column (Waters), and further peptide separation was achieved on a nano-Ultra Performance Liquid Chromatography (UPLC) RP-C18 column (Waters, BEH130 C18 column, 75 μm i.d., 250 mm long) in a nanoACQUITY UPLC system, using a 45 minute linear acetonitrile gradient. The column outlet was directly coupled to an Electrospray ionization (ESI) ion source of a Orbitrap Velos type mass spectrometer (Thermo Scientific, Waltham, USA), operating in a regime of a data-dependent MS to MS/MS switch with HCD-type peptide fragmentation. An electrospray voltage of 1.5 kV was used.

### Bioinformatics

Raw data files were pre-processed with Mascot Distiller software (version 2.4.2.0, MatrixScience). The obtained peptide masses and fragmentation spectra were matched to the National Center Biotechnology Information (NCBI) non-redundant database (115,488,495 sequences/ 42,334,050,411 residues), with a Nematoda filter (748,652 sequences) using the Mascot search engine (Mascot Daemon v. 2.4.0, Mascot Server v. 2.4.1, MatrixScience). The following search parameters were applied: enzyme specificity was set to trypsin, peptide mass tolerance to ± 30 ppm and fragment mass tolerance to ± 0.1 Da. The protein mass was left as unrestricted, and mass values as monoisotopic, with one missed cleavage being allowed. Alkylation of cysteine by carbamidomethylation as fixed, oxidation of methionine was set as a variable modification. Protein identification was performed using the Mascot search engine (MatrixScience), with a probability-based algorithm. The expected value threshold of 0.05 was used for the analysis, which means that all peptide identifications had less than a one-in-20 chance of being a random match. All proteins identified in the MASCOT search were subsequently assigned to the UniProtKB database (https://www.uniprot.org/) and QuickGO (http://www.ebi.ac.uk/QuickGO/) and classified in gene ontology (GO) in accordance with its molecular function, biological process and cellular component information.

## Results

### 2-DE and immunoblot analysis of Ad and ML proteins of *T. britovi*

**Fig. 1:**
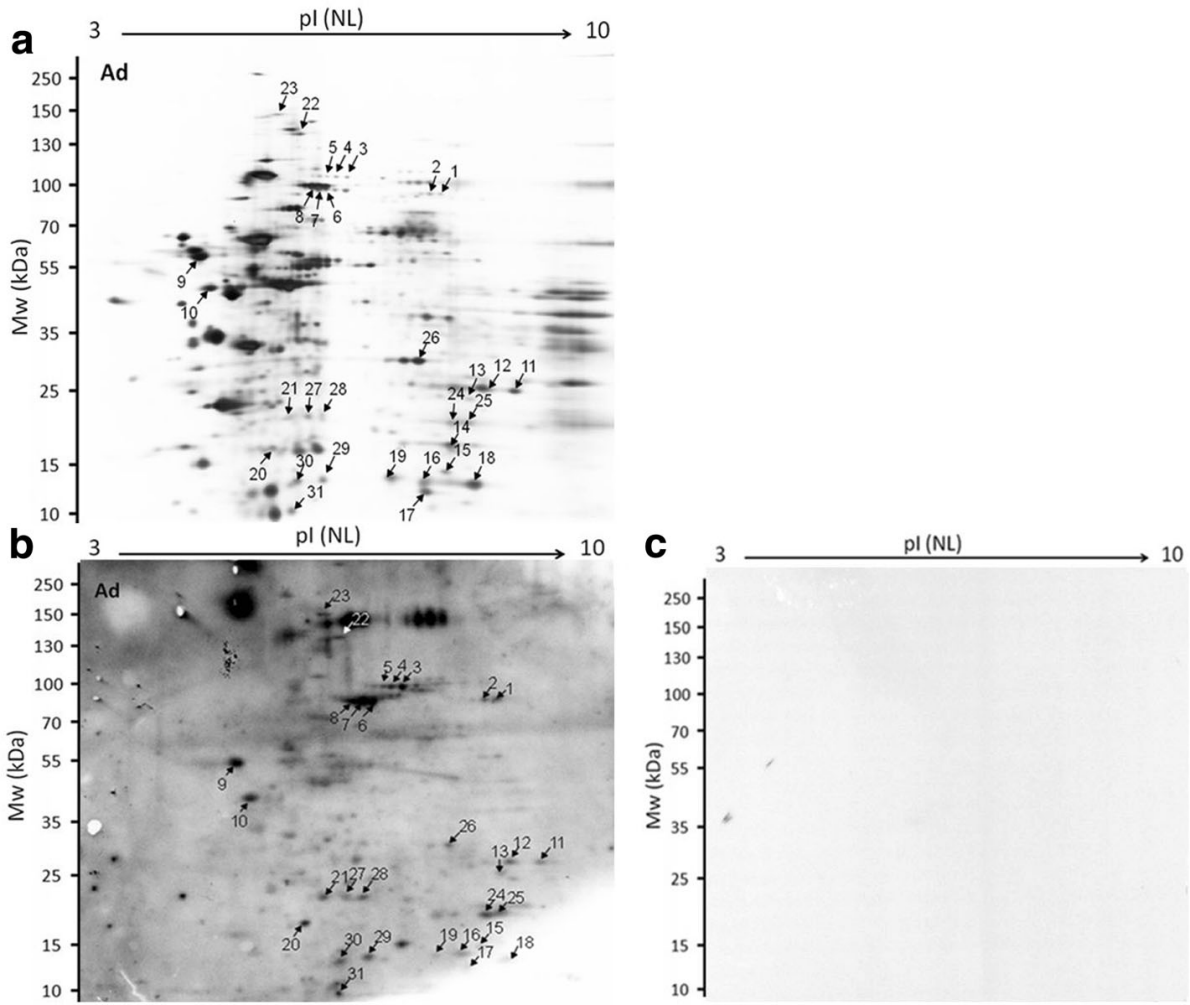
An image of 2-DE separations and immunoblot analysis of somatic antigen extract of *T. britovi* adult worms (Ad). **a** 2-DE gels were stained with a silver stain. **b** 2D-immunoblot of Ad proteins were probed with infected pig sera at 10 dpi. **c** 2D-immunoblot of Ad proteins probed with uninfected swine sera. Matched spots selected for subsequent LC-MS/MS analysis are marked

**Fig. 2:**
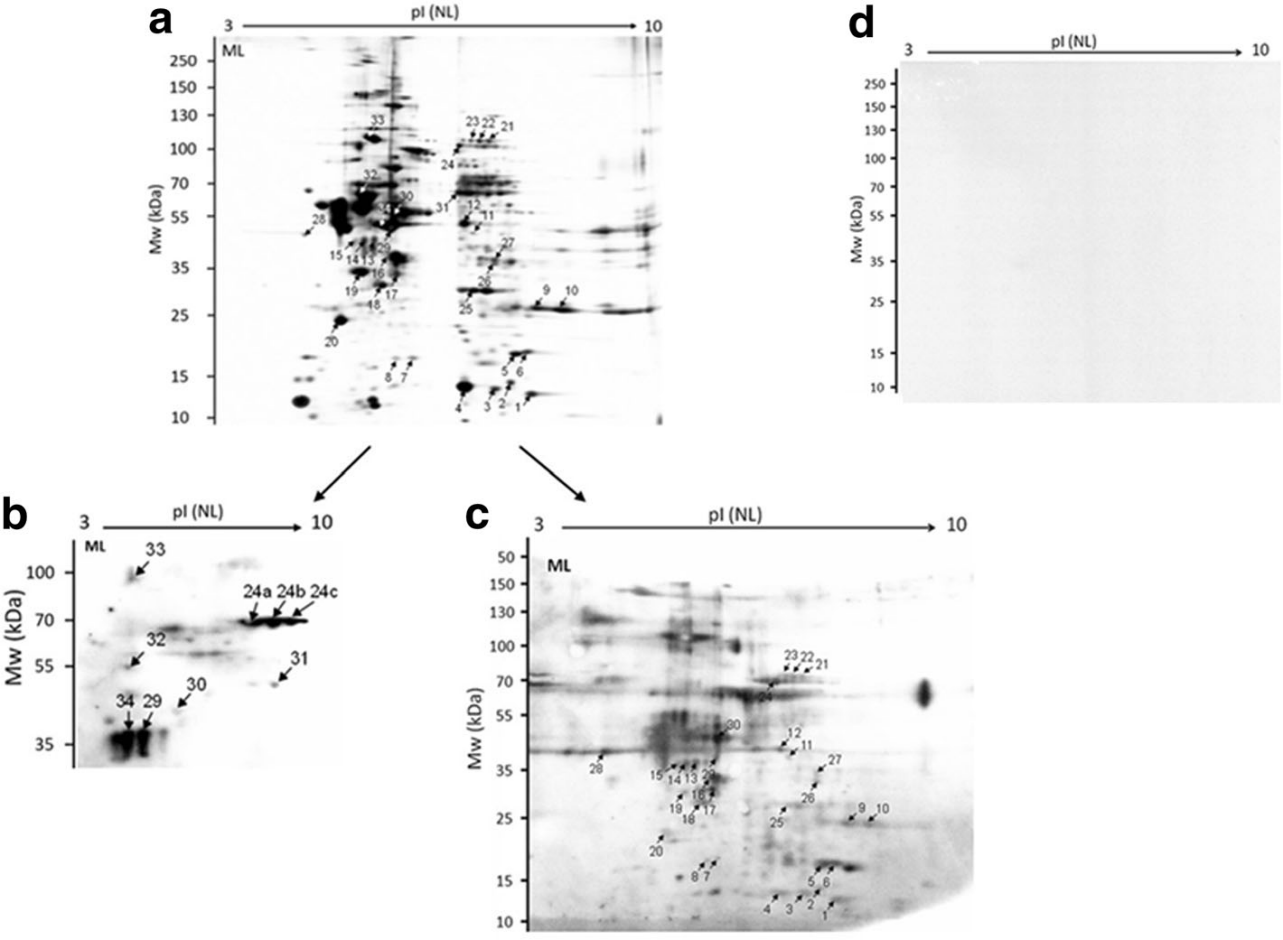
An image of 2-DE separations and immunoblot analysis of somatic antigen extract of *T. britovi* muscle larvae (ML). **a** 2-DE gels were stained with a silver stain. 2D-immunoblot of ML proteins were probed with infected pig sera at 10 dpi (**b**) and at 60 dpi (**c**). **d** 2D- immunoblot of ML proteins probed with uninfected swine sera. Matched spots selected for subsequent LC-MS/MS analysis are marked

To identify species-specific parasite antigens, extracts of *T. britovi* Ad and ML were separated by IEF on 7 cm, pH 3–10 strips. Figures 1a and 2a represent one of the three replicated silver-stained proteome gels used for further analysis. The proteomes of Ad and ML presented 261 and 272 spots, respectively, with a pH range of 3–10 and molecular weight (MW) ranging from 10 kDa to 250 kDa (Figs. 1a, 2a). The results of the 2-DE immunoblot of the Ad and ML extracts are given in Figs. 1b and 2b, c. Approximately 31 Ad-immunoreactive protein spots and nine ML protein spots were positively recognized by *T. britovi*-infected swine sera at 10 dpi. Sera taken from pigs at 60 dpi recognized 30 ML protein spots. Potentially immunogenic proteins migrated with a MW between 10 and 150 kDa (Figs. 1b, 2b, c). These immunoreactive spots matched to the corresponding protein spots on silver stained gels, and were selected for further LC-MS/MS identification. No protein reacted to uninfected swine sera (Figs. 1c, 2d).

### LC-MS/MS analysis of antigenic proteins of *T. britovi* specific for adult worms

The protein data obtained in the present study were compared against deposited protein sequences available for other *Trichinella* spp. The obtained MS/MS datasets were therefore searched against the NCBI database with the Mascot search engine, and the samples detected as *Trichinella* spp.-specific were selected based on score, matches and sequence coverage data. Thirty-one of the positive spots recognized by the *T. britovi*-infected serum samples taken at 10 dpi were matched and located on the silver-stained gels and then subjected to LC-MS/MS analysis (Table 1). The results revealed the presence of 45 proteins with potential antigenic character, among which 29 were specific only for the adult stage of *T. britovi*. Five of these antigenic proteins were present in more than one spot (Table 2 ), and most of the analyzed spots contained more than one protein. The highest number of proteins were identified from spot number 22, containing five proteins, spots 6, 7 and 19 containing four proteins, and spots 8, 23, 28, 30 and 31 containing three proteins (Table 1). Only one protein was present in nine spots (nos 2, 4, 11, 12, 13, 14, 24, 25 and 29). No protein set was found in spot no. 21. Several of the immunogenic proteins specific for adult worms were matched to myosin, actin-depolymerizing factor 1, isoforms a/b, heat-shock cognate 71 kDa protein, stress-70 protein, Rho GDP-dissociation inhibitor 1, paramyosin or serine/arginine-rich splicing factor 1 (Tables 1 and 2).

**Table 1:** Results of LC-MS/MS analysis of *Trichinella britovi* adult worms (Ad) selected spots which reacted with pig sera collected at 10 dpi

Spot	NCBIprot accession no.	MS^a^	MP^b^	Seq^c^	SC (%)^d^	emPAI^e^	Mr(kDa)/pI^f^	Description
1	KRZ04300.1	746	11	10	15	0.82	71.823/6.58	Transketolase, partial
	KRY23714.1	363	5	5	8	0.33	75.949/8.54	Succinate dehydrogenase (ubiquinone) flavoprotein subunit, mitochondrial, partial
2	KRY10810.1	817	13	11	16	1.08	70.999/6.60	Transketolase
3	KRY09282.1	1067	18	17	25	1.86	73.168/6.07	Intermediate filament protein ifa-1
	KRY23083.1	1015	16	16	22	1.19	76.326/6.17	Stress-70 protein, mitochondrial, partial
4	KRY23083.1	405	6	6	10	0.32	76.326/6.17	Stress-70 protein, mitochondrial, partial
5	KRY23083.1	247	3	3	5	0.18	76.326/6.17	Stress-70 protein, mitochondrial, partial
	KRZ06996.1	99	2	2	2	0.12	74.844/5.78	Intermediate filament protein ifa-1, partial
6	KRY11608.1	1889	36	26	40	4.74	75.526/6.24	Intermediate filament protein B
	KRY09282.1	1883	31	27	38	4.10	73.168/6.07	Intermediate filament protein ifa-1
	KRY16427.1	1772	33	22	22	1.78	108.386/6.14	Heat-shock cognate 71 kDa protein, partial
	CAA73574.1	1612	30	21	31	3.15	71.860/5.77	Heat-shock protein 70
7	KRY58599.1	1946	38	26	23	1.54	131.529/6.88	Heat-shock cognate 71 kDa protein, partial
	KRY11608.1	1933	37	23	38	5.03	75.526/6.24	Intermediate filament protein B
	CAA73574.1	1773	32	25	36	3.87	71.860/5.77	Heat-shock protein 70
	KRY09282.1	1503	25	21	29	2.95	73.168/6.07	Intermediate filament protein ifa-1
8	CAA73574.1	1633	35	22	33	3.42	71.860/5.77	Heat-shock protein 70
	KRY11608.1	1094	18	17	25	1.62	75.526/6.24	Intermediate filament protein B
	KRZ06996.1	906	13	12	17	0.98	74.844/5.78	Intermediate filament protein ifa-1, partial
9	KRY21440.1	646	12	9	16	0.97	62.134/5.04	Calreticulin
	KRY19442.1	329	5	5	15	0.81	35.572/9.61	Y-box factor -like protein
10	KRY21426.1	507	8	7	27	1.80	29.821/4.64	Myosin light chain kinase, smooth muscle
	AET09716.1	215	3	3	15	0.78	22.620/4.54	Tropomyosin, partial
11	KRZ13803.1	412	6	5	17	1.07	35.565/8.61	32 kDa beta-galactoside-binding lectin lec-3 (Galectin)
12	KRY20423.1	465	8	6	21	1.48	33.995/7.69	32 kDa beta-galactoside-binding lectin (Galectin)
13	KRX13351.1	62	1	1	4	0.15	29.477/8.21	RNA-binding protein rnp-1
14	KRX46812.1	497	7	6	32	2.26	22.941/7.07	Peroxiredoxin-2, partial
15	KRZ77496.1	378	6	4	3	0.08	23.7876/6.48	Dedicator of cytokinesis protein 1
	KRY20040.1	140	3	2	10	0.97	18.977/6.97	Heat-shock protein beta-1
16	KRY20040.1	445	12	6	34	3.69	18.977/6.97	Heat-shock protein beta-1
	KRX20324.1	323	6	4	28	1.33	19.886/8.12	OV-16 antigen, partial
17	KRY20040.1	405	11	5	30	3.01	18.977/6.97	Heat-shock protein beta-1
	KRX20324.1	369	6	4	28	1.43	19.886/8.12	OV-16 antigen, partial
18	KRX47621.1	701	19	9	53	7.94	18.951/6.97	Heat-shock protein beta-1
	KRX19442.1	106	2	2	10	0.57	18.490/8.74	Transcription factor BTF3 -like protein 4
19	KRX16844.1	358	11	6	30	2.86	18.894/6.32	Alpha-crystallin B chain
	KRY20040.1	297	6	4	26	2.06	18.977/6.97	Heat-shock protein beta-1
	KRZ07637.1	278	3	3	14	0.75	23.026/6.12	Stromal cell-derived factor 2
	KRZ08373.1	75	2	2	9	0.47	22.324/7.68	Peroxiredoxin-2
20	KRX18074.1	398	8	6	9	0.74	54.209/4.92	BAG family molecular chaperone regulator 2, partial
	KRZ17076.1	165	3	2	10	0.75	22.920/5.45	Rho GDP-dissociation inhibitor 1
21	Unidentified	–	–	–	–	–	–	–
22	KRY31449.1	1224	26	15	20	1.11	91.608/5.31	Transitional endoplasmic reticulum ATPase -like protein 2
	KRZ08767.1	1011	13	13	7	0.27	234.755/5.91	Myosin-4, partial
	KRZ03705.1	287	3	3	4	0.13	101.672/5.38	Paramyosin
	KRY00202.1	206	4	3	5	0.20	92.727/5.26	Heat-shock 70 kDa protein 4L
	KRY14731.1	172	3	3	3	0.10	131.892/6.63	CAP-Gly domain-containing linker protein 1
23	KRY18882.1	994	15	12	14	0.79	108.605/5.51	LIM domain and actin-binding protein 1
	KRZ08767.1	345	6	6	3	0.12	234.755/5.91	Myosin-4, partial
	KRZ13693.1	302	5	5	4	0.20	125.148/6.31	Integrin alpha pat-2
24	KRZ06959.1	252	3	3	11	0.61	28.479/7.74	Triosephosphate isomerase, partial
25	KRZ12367.1	299	4	4	24	1.19	24.385/7.01	GTP-binding nuclear protein Ran
26	KRX15368.1	656	14	10	33	3.52	34.025/6.29	32 kDa beta-galactoside-binding lectin, partial
	KRY59871.1	115	2	2	12	0.32	30.478/9.08	Serine/arginine-rich splicing factor 1, partial
	KRX23478.1	101	2	2	4	0.27	35.530/6.82	Protein MEMO1, partial
27	KRX41818.1	376	5	5	21	1.13	29.431/5.58	Putative phosphomannomutase
	KRY00151.1	137	3	2	4	0.25	59.844/5.54	ATP synthase subunit beta, mitochondrial
	KRY00848.1	114	2	2	2	0.09	108.425/6.27	Heat-shock 70 kDa protein, partial
28	KRX20997.1	368	6	6	8	0.40	76.565/8.34	Guanine nucleotide-binding proteinalpha-12 subunit, partial
	KRX16428.1	294	6	5	23	1.65	26.141/5.80	V-type proton ATPase subunit E
	KRZ10894.1	177	3	3	9	0.58	27.709/7.55	GrpE -like protein 1, mitochondrial
29	KRY01036.1	394	8	5	27	2.16	22.274/6.88	Actin-depolymerizing factor 1, isoforms a/b, partial
30	KRY01036.1	440	8	5	27	2.12	22.274/6.88	Actin-depolymerizing factor 1, isoforms a/b, partial
	KRY17912.1	295	4	4	18	1.63	17.440/6.18	Uncharacterized protein T12_13420
	KRY00151.1	143	4	2	4	0.33	59.844/5.54	ATP synthase subunit beta, mitochondrial
31	KRY01216.1	278	5	5	21	1.85	19885/5.43	Ubiquitin-conjugating enzyme E2 G1, partial
	KRY21297.1	199	4	3	14	1.15	21.790/8.89	Peptide methionine sulfoxide reductase MsrB
	KRY15966.1	185	4	3	10	0.44	34.259/6.31	Hypothetical protein T12_8663

^a^Mascot score

^b^Matched peptide

^c^Sequence

^d^Sequence coverage (%)

^e^Exponentially modified protein abundance index

^f^Experimental nominal mass (kDa) and isoelectric point

**Table 2:** Alphabetical list of stage-specific antigenic proteins of adult worms of *T. britovi*, which reacted with pig sera collected at 10 dpi, together with spot number information. Identification by LC-MS/MS

Protein name	Spot number
Actin-depolymerizing factor 1, isoforms a/b, partial	29, 30
BAG family molecular chaperone regulator 2, partial	20
Calreticulin	9
CAP-Gly domain-containing linker protein 1	22
GrpE-like protein 1, mitochondrial	28
Guanine nucleotide-binding protein alpha-12 subunit, partial	28
Heat-shock 70 kDa protein 4L	22
Heat-shock cognate 71 kDa protein, partial	6, 7
Hypothetical protein T12_8663	31
Integrin alpha pat-2	23
LIM domain and actin-binding protein 1	23
Myosin-4, partial	22, 23
Myosin light chain kinase, smooth muscle	10
Paramyosin	22
Peptide methionine sulfoxide reductase MsrB	31
Putative phosphomannomutase	27
Rho GDP-dissociation inhibitor 1	20
RNA-binding protein rnp-1	13
Serine/arginine-rich splicing factor 1, partial	26
Stress-70 protein, mitochondrial	3, 4, 5
Stromal cell-derived factor 2	19
Succinate dehydrogenase (ubiquinone) flavoprotein subunit, mitochondrial, partial	1
Transitional endoplasmic reticulum ATPase -like protein 2	22
Transketolase /partial	1, 2
Triosephosphate isomerase, partial	24
Ubiquitin-conjugating enzyme E2 G1, partial	31
Uncharacterized protein T12_13420	30
Y-box factor -like protein	9
V-type proton ATPase subunit E	28

### LC-MS/MS analysis of antigenic proteins of *T. britovi* specific for muscle larvae

Nine ML protein spots cross-reacting with *T. britovi* infected swine sera were identified by MS analysis at 10 dpi, and 30 spots were found at 60 dpi (Tables 3 and 4).

**Table 3:** Results of LC-MS/MS analysis of *Trichinella britovi* muscle larvae (ML) selected spots which reacted with pig sera collected at 10 dpi

Spot		NCBIprot accession No.	MS^a^	MP^b^	Seq^c^	SC (%)^d^	emPAI^e^	Mr(kDa)/pI^f^	Description
24	a	KRY11608.1	1765	36	26	36	4.01	75.526/6.24	Intermediate filament protein B
		KRY09282.1	1457	30	22	30	2.92	73.168/6.07	Intermediate filament protein ifa-1
	b	KRY11608.1	1903	40	29	39	5.11	75.526/6.24	Intermediate filament protein B
		KRY09282.1	1541	33	23	33	3.30	73.168/6.07	Intermediate filament protein ifa-1
	c	KRY11608.1	930	14	13	19	1.24	75.526/6.24	Intermediate filament protein B
		KRY09282.1	2328	66	34	47	7.99	73.168/6.07	Intermediate filament protein ifa-1
29		XP_003373575.1	1206	52	15	41	4.74	42.210/5.30	Actin-5C
30		XP_003373575.1	527	9	8	25	1.47	42.210/5.30	Actin-5C
		KRY50178.1	415	8	6	15	0.89	46.783/5.44	Hypothetical protein T03_17187
		KRZ06996.1	160	3	3	4	0.12	74.844/5.78	Intermediate filament protein ifa-1, partial
		KRZ09733.1	323	5	5	5	0.28	96.031/6.00	Mitochondrial-processing peptidase subunit beta, partial
		KRX47705.1	293	4	4	3	0.14	150.442/6.28	Serine protease 30
31		KRZ02603.1	1083	28	14	34	2.98	50.922/6.01	Enolase, partial
		KRY13126.1	544	10	9	20	1.47	48.623/5.41	26S protease regulatory subunit 7
32		KRY18793.1	883	19	14	30	3.45	54.997/5.00	Protein disulfide-isomerase 2
		OUC40875.1	749	17	10	26	2.73	48.387/4.87	Putative Tubulin/FtsZ family, GTPase domain protein
		KRY00151.1	655	14	8	17	1.31	59.844/5.54	ATP synthase subunit beta, mitochondrial
33		KRX41020.1	1127	27	16	27	1.92	72.856/5.09	Heat-shock 70 kDa protein C, partial
		KRY00702.1	364	6	5	9	0.46	68.894/5.08	V-type proton ATPase catalytic subunit A
34		Unidentified	–	–	–	–	–	–	–

^a^Mascot score

^b^Matched peptide

^c^Sequence

^d^Sequence coverage (%)

^e^Exponentially modified protein abundance index

^f^Experimental nominal mass (kDa) and isoelectric point

**Table 4:** Results of LC-MS/MS analysis of *Trichinella britovi* muscle larvae (ML) selected spots which reacted with pig sera collected at 60 dpi

Spot	NCBIprot accession No.	MS^a^	MP^b^	Seq^c^	SC (%)^d^	emPAI^e^	Mr(kDa)/pI^f^	Description
1	KRX47621.1	732	22	10	53	13.42	18.951/6.97	Heat-shock protein beta-1
	KRX19442.1	133	2	2	10	0.64	18.490/8.74	Transcription factor BTF3 -like protein 4
2	KRX14469.1	241	3	3	2	0.06	247.333/6.85	Dedicator of cytokinesis protein 1
	KRZ13097.1	165	5	3	13	1.75	19.090/5.43	Heat-shock protein beta-1, partial
3	KRY20040.1	376	8	6	34	4.92	18.977/6.97	Heat-shock protein beta-1
	KRX20324.1	310	5	4	28	1.64	19.886/8.12	OV-16 antigen, partial
4	KRX16844.1	662	60	10	66	10.33	18.894/6.32	Alpha-crystallin B chain
	KRY20040.1	396	8	7	35	4.78	18.977/6.97	Heat-shock protein beta-1
	KRY18783.1	109	2	2	9	0.18	25.119/6.44	Stromal cell-derived factor 2
5	KRX46812.1	546	11	8	41	4.02	22.941/7.07	Peroxiredoxin-2, partial
6	KRX46812.1	409	8	6	28	2.07	22.941/7.07	Peroxiredoxin-2, partial
	KRZ12367.1	292	4	4	19	1.02	24.385/7.01	GTP-binding nuclear protein Ran
	KRX18658.1	226	3	3	15	0.73	23.516/6.92	ATP synthase subunit d, mitochondrial
7	Unidentified	–	–	–	–	–	–	–
8	Unidentified	–	–	–	–	–	–	–
9	KRZ13803.1	406	7	5	17	1.11	35.565/8.61	32 kDa beta-galactoside-binding lectin lec-3 (Galectin)
10	KRZ13803.1	584	9	7	24	1.58	35.565/8.61	32 kDa beta-galactoside-binding lectin lec-3 (Galectin)
	KRY30017.1	304	6	5	17	0.82	34.995/8.74	Putative 3-hydroxyacyl-CoA dehydrogenase
11	KRZ13161.1	105	2	2	6	0.23	42.112/7.12	Glutamine synthetase
12	KRY11984.1	432	7	7	15	0.90	49.560/6.59	Poly-cysteine and histidine-tailed protein
	KRX28313.1	364	7	6	14	1.01	45.667/6.09	Calponin -like protein OV9M, partial
	KRX47308.1	240	3	3	3	0.14	107.151/6.52	Deoxyribonuclease-2-alpha
13	KRY01407.1	324	4	4	10	0.46	51.099/5.91	Cuticlin-1, partial
14	KRY01407.1	319	4	4	10	0.48	51.099/5.91	Cuticlin-1, partial
	KRY00848.1	166	3	3	2	0.15	108.425/6.27	Heat-shock 70 kDa protein, partial
15	CBX25713.1	322	5	5	14	1.14	32.896/4.65	Tropomyosin, partial
16	KRY09099.1	476	8	8	22	1.47	38.218/5.20	Hypothetical protein T12_13379, partial
	KRX15676.1	174	3	3	7	0.43	35.904/5.46	40S ribosomal protein SA, partial
17	KRY09099.1	381	6	6	16	1.20	38.218/5.20	Hypothetical protein T12_13379, partial
	KRZ15717.1	217	4	3	9	0.46	39.852/5.63	Guanine nucleotide-binding protein subunit beta-1, partial
	KRY18502.1	203	4	3	3	0.26	65.700/4.95	Microtubule-associated protein RP/EB family member 3, partial
	KRX15059.1	126	3	2	5	0.52	36.189/5.00	Disorganized muscle protein 1
18	KRX21567.1	504	11	6	21	1.66	40.427/5.49	Pyruvate dehydrogenase E1 component subunit beta, mitochondrial
19	KRX15059.1	667	23	9	28	2.96	36.189/5.00	Disorganized muscle protein 1
	KRZ03570.1	403	6	6	18	1.20	34.457/4.75	Tropomyosin
20	XP_003378934.1	1001	20	12	46	6.93	28.294/4.83	14-3-3 protein zeta
	AET09716.1	248	4	4	19	1.09	22.620/4.54	Tropomyosin, partial
	KRX19348.1	159	3	3	13	0.56	28.034/4.82	Toll-interacting protein
21	KRZ50222.1	917	16	14	3	0.17	449.723/6.87	Propionyl-CoA carboxylase alpha chain, mitochondrial
22	KRZ50222.1	1081	18	17	4	0.19	449.723/6.87	Propionyl-CoA carboxylase alpha chain, mitochondrial
23	KRY09873.1	557	9	9	2	0.09	441.173/6.76	Propionyl-CoA carboxylase alpha chain, mitochondrial
24	KRY45949.1	1999	45	29	42	6.80	73.429/6.07	Intermediate filament protein ifa-1
	KRY09282.1	1720	36	26	36	4.01	75.526/6524	Intermediate filament protein B
25	KRX15368.1	437	7	7	20	1.80	34.025/6.29	32 kDa beta-galactoside-binding lectin, partial
	KRZ10402.1	91	2	2	4	0.33	35.568/6.67	Protein MEMO1, partial
	KRZ78587.1	42	3	1	2	0.11	48.445/5.59	Secernin-3
26	KRY07641.1	341	6	5	18	1.14	38.033/6.38	1,5-anhydro-D-fructose reductase
27	KRY07641.1	239	4	4	12	0.63	38.033/6.38	1,5-anhydro-D-fructose reductase
28	Unidentified	–	–	–	–	–	–	–
29	XP_003373575.1	1255	39	15	41	4.48	42.210/5.30	Actin-5C
	AET09716.1	168	2	2	11	0.45	22.620/4.54	Tropomyosin, partial
	KRY38295.1	160	3	3	6	0.26	54.444/6.39	Secernin-3
	KRZ06996.1	232	3	3	4	0.19	74.844/5.78	Intermediate filament protein ifa-1, partial
30	KRY50178.1	993	18	15	40	4.11	46.783/5.44	Hypothetical protein T03_17187
	XP_003373575.1	527	9	8	25	1.47	42.210/5.30	Actin-5C
	KRZ06996.1	363	6	6	9	0.47	74.844/5.78	Intermediate filament protein ifa-1, partial
	KRX47705.1	293	4	4	3	0.14	150.442/6.28	Serine protease 30
	KRZ09733.1	323	5	5	5	0.28	96.031/6.00	Mitochondrial-processing peptidase subunit beta, partial
	KRZ17128.1	256	4	4	9	0.51	46.607/5.26	Putative histone-binding protein Caf1
	KRY13378.1	250	5	5	9	0.54	54.969/5.66	Rab GDP dissociation inhibitor alpha

^a^Mascot score

^b^Matched peptide

^c^Sequence

^d^Sequence coverage (%)

^e^Exponentially modified protein abundance index

^f^Experimental nominal mass (kDa) and isoelectric point

LC-MS/MS analysis revealed the presence of 13 immunoreactive ML proteins recognized by sera at 10 dpi samples, nine of which were stage-specific (Table 5). In the samples at 60 dpi, 39 proteins were recognized by sera, with only 25 being stage-specific (Table 6). One protein recognized by sera at 10 dpi was present in two spots (nos 29, 30) (Table 5) and seven proteins recognized by sera at 60 dpi were present in more than one spot (Table 6). The highest number of proteins, i.e. seven, were observed in spot number 30, followed by four proteins in spots 17 and 29, and three proteins in spots 4, 6, 12, 20, 30 and 32 (Table 4 ). The remaining spots contained fewer than three proteins (Tables 3 and 4). Only spot no 34 contained no proteins recognized by sera at 10 dpi, while at 60 dpi, three spots contained no recognized proteins (7, 8 and 28) (Tables 3 and 4).

**Table 5:** Alphabetical list of stage-specific antigenic proteins of muscle larvae of *T. britovi*, which reacted with pig sera collected at 10 dpi, together with spot number information. Identification by LC-MS/MS

Protein name	Spot number
26S protease regulatory subunit 7	31
Actin-5C	29/30
Enolase, partial	30
Hypothetical protein T03_17187	30
Protein disulfide-isomerase 2	32
Putative Tubulin/FtsZ family, GTPase domain protein	32
V-type proton ATPase catalytic subunit A	33
Mitochondrial-processing peptidase subunit beta, partial	30
Serine protease 30	30

**Table 6:** Alphabetical list stage-specific antigenic proteins of muscle larvae of *T. britovi*, which reacted with pig sera collected at 60 dpi, together with spot number information. Identification by LC-MS/MS

Protein name	Spot number
1,5-anhydro-D-fructose reductase	26, 27
14-3-3 protein zeta	20
40S ribosomal protein SA, partial	16
Actin-5C	29, 30
ATP synthase subunit d, mitochondrial	6
Calponin -like protein OV9M, partial	12
Cuticlin-1, partial	13, 14
Deoxyribonuclease-2-alpha	12
Disorganized muscle protein 1	17, 19
Glutamine synthetase	11
Guanine nucleotide-binding protein subunit beta-1, partial	17
Hypothetical protein T03_17187	30
Hypothetical protein T12_13379, partial	16, 17
Microtubule-associated protein RP/EB family member 3, partial	17
Mitochondrial-processing peptidase subunit beta, partial	30
Poly-cysteine and histidine-tailed protein	12
Propionyl-CoA carboxylase alpha chain, mitochondrial	21, 22, 23
Putative 3-hydroxyacyl-CoA dehydrogenase	10
Putative histone-binding protein Caf1	30
Pyruvate dehydrogenase E1 component subunit beta, mitochondrial	18
Rab GDP dissociation inhibitor alpha	30
Secernin-3	25, 29
Serine protease 30	30
Stromal cell-derived factor 2	4
Toll-interacting protein	20

The following immunogenic proteins specific for the ML stage were identified in the 10 dpi serum samples: 26S protease regulatory subunit 7; actin-5C; enolase; protein disulfide-isomerase 2; V-type proton ATPase catalytic subunit A; and serine protease 30 (Table 5). The following were identified in the 60 dpi samples: 14-3-3 protein zeta; 40S ribosomal protein SA; calponin-like protein OV9M; propionyl-CoA carboxylase alpha chain; Rab GDP dissociation inhibitor alpha; secernin-3; serine protease 30; Toll-interacting protein (Table 6). Finally, the following proteins were identified in both the 10 and 60 dpi samples: actin 5C; serine protease; intermediate filament protein (IFA-1); and mitochondrial-processing peptides subunit beta (Tables 5 and 6).

### LC-MS/MS analysis of antigenic proteins common for both stages of *T. britovi*

Although some proteins were found to be specific for both the Ad and ML stages of *T. britovi*, most were common to both stages (Table 7). The following proteins appeared in both proteomes, and were most frequently identified from multiple spots: heat-shock protein beta-1 (present in five spots - Ad 10 dpi, four spots - ML 60 dpi); intermediate filament protein IFA-1; partial (present in five spots - Ad 10 dpi, three spots - ML 60 dpi, four spots - ML 10 dpi); intermediate filament protein IFA-1 (present in five spots - Ad 10 dpi, three spots - 10 dpi and one spot - ML 60 dpi); peroxiredoxin-2/partial (present in three spots - Ad 10 dpi, two spots - ML 60 dpi); tropomyosin (present in one spot - Ad 10 dpi, four spots - ML 60 dpi); and heat-shock 70 kDa protein (present in four spots - Ad 10 dpi, one spot - ML 60 dpi) (Tables 1, 3, 4 and 7). The presence of these different isoforms could be attributed to differences in amino acid sequence, alternative splicing or post-translational modifications. The dominant proteins for both stages were identified as heat-shock protein 70 kDa, heat-shock protein beta-1, intermediate filament B and IFA-1 (Table 7).

**Table 7:** Alphabetical list of antigenic proteins, common for both adult worms (Ad) and muscle larvae (ML) stages *T. britovi* recognized by sera at 60 dpi and 10 dpi, together with spot number information. Identification by LC-MS/MS

Protein name	Spot number Ad *T. britovi*	Spot number ML *T. britovi*	
	10 dpi	10 dpi	60 dpi
32 kDa beta-galactoside-binding lectin lec-3 (Galectin)	11	–	9, 10
32 kDa beta-galactoside-binding lectin, partial (Galectin)	12, 26	–	25
Alpha-crystallin B chain	19	–	4
ATP synthase subunit beta, mitochondrial	27, 30	32	–
Dedicator of cytokinesis protein 1	15	–	2
GTP-binding nuclear protein Ran	25	–	6
Heat-shock 70 kDa protein, partial	6, 7, 8, 27	–	14
Heat-shock protein beta-1	15, 16, 17, 18, 19	–	1, 2, 3, 4
Intermediate filament protein B	6, 7, 8	24 a,b,c	24
Intermediate filament protein ifa-1, partial	3, 5, 6, 7, 8	24a/b/c, 30	24, 29, 30
OV-16 antigen, partial	16, 17	–	3
Peroxiredoxin-2, partial	14, 19, 30	–	5, 6
Protein MEMO1, partial	26	–	25
Transcription factor BTF3 -like protein 4	18	–	1
Tropomyosin, partial	10	–	15, 19, 20, 29
V-type proton ATPase subunit E	28	33	–

### Gene ontology (GO) analysis

The gene ontology (GO) database was used to identify the antigenic proteins of the Ad and ML stages according to their molecular function, cellular component and biological process.

**Fig. 3:**
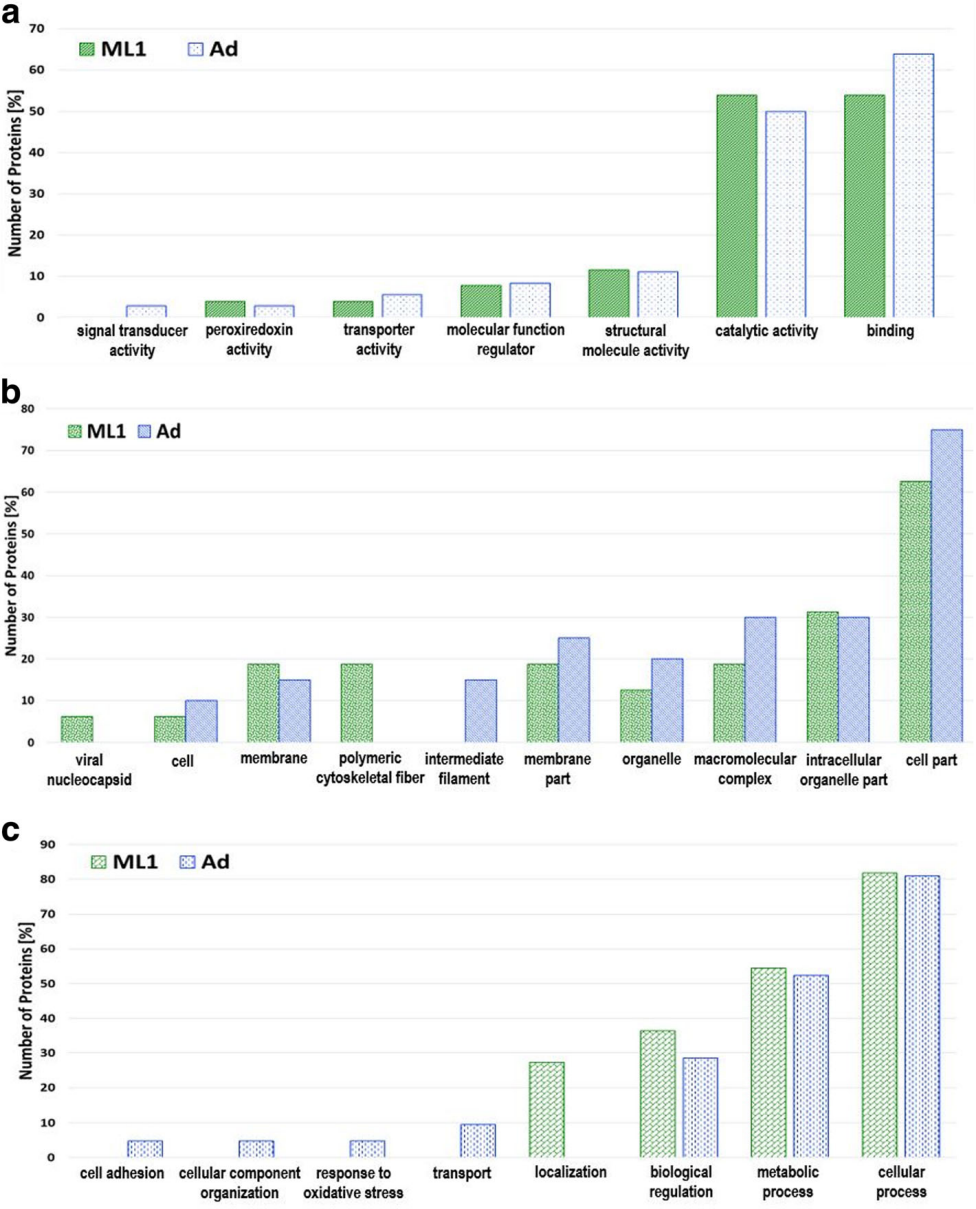
Comparison of Gene Ontology (GO) database analysis outcomes for *T. britovi* muscle (ML) and adult (Ad) larvae identified proteins. The proteins were categorized according to molecular function (**a**), cellular component (**b**) and biological process (**c**)

For the *T. britovi* adult stage, the proteins were classified according to molecular function (39), cellular components (21) and biological process (21). Seven subcategories of molecular function were determined, the most abundant of which were binding (24) and catalytic activity (18); however, structural molecule activity (6), molecular function regulation (3), transporter activity (2), signal transducer (1) or peroxiredoxin activity (1) subcategories were also observed. Eight subcategories for cellular component were determined, the most numerous being the cell part subcategory (18); however, intracellular organelle part (7), macromolecular complex (7), organelle (6), membrane part (5), intermediate filament (4), membrane (3) or cell (1) subcategories were also observed to a lesser extent. Seven subcategories of biological process were determined. The most abundant were assigned to the cellular process (16) and the metabolic process (11) subcategories, while the remainder were assigned to biological regulation (5), localization (3), transport (2), response to oxidative stress (1), cell adhesion (1) or cellular component organization (1) (Fig. 3a-c). Based on the gene ontology analysis, the potentially antigenic proteins of *T. britovi* muscle larvae which reacted with both 10 dpi and 60 dpi pig sera, were categorized according to molecular function (35), cellular component (24) or biological process (18). Six subcategories for molecular function were determined. The most abundant were binding (20), and catalytic activity (20), whereas structural molecule activity (6), transmembrane transporter activity (3), molecular function regulation (2) or peroxiredoxin activity (1) were visibly less numerous. Eight cellular component subcategories were determined, with the most numerous subcategory being cell part (18), followed by intracellular organelle part (9), macromolecular complex (7), polymeric cytoskeletal fiber (6), membrane part (5), membrane (3), organelle (3) and cell (1). Four subcategories for biological process were determined. The cellular process (15) subcategory was the most numerous, followed by metabolic process (11), biological regulation (5) and localization (5) (Fig. 3a-c).

## Discussion

Recent reports indicate most cases of *Trichinella britovi* infection occur amongst patients unaware of eating improperly cooked meat products [32–34, 38]. Early diagnosis of trichinellosis is crucial, as anthelmintic drug treatment is much more effective if administered during the initial phases before muscle larvae become encapsulated [39].

In trichinellosis, the interaction between the parasite and the host is influenced by the *Trichinella* life-cycle, which includes a range of stage-specific antigens, immune evasion strategies and modulatory effects on host responses. The combination of immunoblot analysis and proteomic techniques, such as the two-dimensional gel electrophoresis and mass spectrometry used in the present study, is a comprehensive approach to identifying *Trichinella* proteins [40]. Although most proteomic studies have focused on the identification of proteins characteristic of *T. spiralis* life-cycle stages, same of them were dedicated to other *Trichinella* species/genotypes including *T. pseudospiralis*, *T. nativa*, *T. papuae* and T8 [18, 19, 21, 26, 41].

However, further effort is still needed to identify the *T. britovi* proteins that may play an important role in understanding host-parasite interactions, and to develop immunological diagnostic methods. Only two papers have addressed the identification of antigenic proteins from *T. britovi*, the second-most common species of *Trichinella* that may affect human health [9, 30]. Dea-Ayuela & Bolaz-Fernandez [30], using 2-DE immunoblot, identified the *T. britovi* proteins that likely belong to the *Trichinella* TSL-1 group of antigens: enolase; P49 antigen; and actins. These proteins play a part in parasite invasion and migration through the host cells. Other studies based on the immunoproteomics of the excretory-secretory systems of *T. britovi* muscle larvae identified a range of proteins, including various glycoproteins (gp43, p49), serine-protease and 5'-nucleotidase [9], that play a role in the development and migration of NBL in host tissue and in the regulation of the immune response by modulating nucleotide levels during infection [42].

The purpose of the present study, therefore, was to identify the *T. britovi*-specific immunodominant proteins present in adult worms and muscle larvae. The crude protein extracts of both stages were separated by 2-DE, subjected to immunoblot analysis with sera from animals infected with *T. britovi* (at 10 dpi and 60 dpi), and identified by LC-MS/MS. A previous immunoproteomic study performed on *T. spiralis* antigens showed that 64 proteins from adult worm crude extract were recognized by sera from pigs and mice infected with *T. spiralis* at 7 dpi, but only seven proteins in muscle larvae crude extract were detected using sera from *T. spiralis*-infected mice and pigs at 5 dpi and 45 dpi, respectively [11, 15, 27].

In the present study, the immunogenic spots recognized by the various pig *T. britovi*-infected sera were divided into three groups according to immunoblotting pattern and LC-MS/MS results: adult (Ad) stage-specific proteins; muscle larvae (ML) stage-specific proteins; and proteins common to both developmental stages. Forty-five proteins in the Ad samples (29 stage-specific for Ad and 16 common) and 13 proteins in the ML samples (9 stage-specific for ML and 4 common) cross-reacted with sera at 10 dpi, while 39 proteins in the ML samples (25 stage-specific for ML and 14 common) reacted with the sera taken at 60 dpi.

Additionally, to further understand the functions of the *T. britovi* proteins, these proteins were categorized according to the GO into biological processes, molecular function and cellular components. The results reveal the presence of a range of proteins known to be antigens involved in the mechanisms of invasion of host tissue and cells, larval migration or molting, immune modulation, metabolic processes in other helminths: actin; heat-shock proteins; paramyosin; 14-3-3-protein; myosin; serine protease; enolase; poly-cysteine and histidine-tailed protein; and deoxyribonuclease-2-alpha [21, 26, 27, 43–45]. Of these proteins, the following were common for both tested *T. britovi* stages: 32 kDa beta galactoside-binding lectin lec-3 (Galectin); heat-shock 70 kDa protein; heat-shock protein beta-1; intermediate filament protein IFA-1; intermediate filament protein B; GTP-binding nuclear protein Ran; OV-16 antigen; protein MEMO1; transcription factor BTF3-like protein 4; tropomyosin; and peroxiredoxin-2. These have previously been found to be present and active throughout the parasite development process; however, they were present in varying amounts, as indicated by the observed dissimilarities in spot intensities.

Adult *T. britovi* are frequently found to contain proteins involved in structural and motor activity, such as myosin-4, myosin light chain kinase, paramyosin, intermediate filament protein B, actin-depolymerizing factor 1 and calreticulin. These cytoskeleton proteins with an actin binding function, are responsible for cellular component organization and actin filament depolymerization, thus facilitating the parasite growth and development processes. Some of them, including actin-depolymerizing factor 1 and paramyosin, were identified in the ML stage but not the early stage of *Trichinella* development [11, 46]. One of these, carleticulin, belongs to the carleticulin family of proteins, which are involved in the protein folding process, and were recently reported to facilitate *T. spiralis* immune evasion by interacting with the first component of the human classical complement pathway, C1q [47]. In addition to its role in muscle length and stability determination, paramyosin also possess immunomodulatory functions. The surface-exposed paramyosin is thought to act as a protective agent during the host inflammatory processes by inhibiting the complement activation cascade and membrane attack complex (MAC) formation [48]. However, V-type proton ATPase subunit E, a member of the ATPase protein family, is activated at a wide pH range and possesses interesting properties under certain biochemical conditions. ATPases are involved in metabolite movements, purging of toxins and energy generation for metabolic processes; they also take part in the environmental response [49, 50] and hence are thought to be involved in the nematode immune response course. Most of the analyzed *T. britovi* antigens are derived from the muscle stage of the larvae. GO analysis of the obtained results showed that some of the proteins participate in various cellular and metabolic processes mostly associated with the synthesis and degradation of macromolecules (nucleotides, proteins) which play an important role in the invasion and development of *Trichinella* in the host [10, 26, 28, 51]. The most frequently identified immunodominant antigens of ML *T. britovi* recognized by infection sera include 14-3-3 protein zeta, actin-5C, ATP synthase subunit d, deoxyribonuclease-2-alpha, poly-cysteine and histide-tailed protein, enolase, V-type proton ATPase catalytic and serine protease 30. For example, the actin-5c protein (recognized by sera at 10 dpi/60 dpi), known to bind ATP molecules (GO), has previously been identified with the use of early and late infection sera [26, 52]. This protein is related to the invasion of a parasite into the intestinal epithelial cells and plays a critical role in larval development [53]. Serine protease 30, with peptidase and hydrolase activities, was recognized by sera at 10 dpi/60 dpi. The protein belongs to serine protease family, along with enzymes that take part in digestion, blood coagulation and fibrinolysis processes. It is involved in host tissues and cell invasions, and plays a pivotal role in nematode molting [54]. Additionally, deoxyribonuclease 2-alpha of the deoxyribonuclease II family was identified, which plays an important role in *Trichinella* invasion, development and survival [55]. The 60 dpi sera also identified the 14-3-3 protein. This is a key regulator of multiple biological processes, including signal transduction, cell differentiation and cell survival, it is also known to induce humoral and cellular immune response and has been tested as a potential vaccine target [56, 57]. The GO analysis revealed that some of the isolated proteins possess catalytic, ligase, hydrolase and peptidase activities, and are responsible for ATP and glutamine synthesis processes; these include ATP-synthase subunit d, glutamine synthase and propionyl-CoA carboxylase alpha chain, all of which were recognized in the 60 dpi sera. GO analysis also showed mitochondrial-processing peptidase (MPP) subunit beta, secernin-3 protein and the previously mentioned serine protease 30 to demonstrate proteolytic and peptidase activity [58]. Microtubule-associated protein RP/EB family member 3 and cuticlin-1, classified as a cellular component belonging to the ML proteome and recognized by sera at 60 dpi, possesses a microtubule binding function. In *Caenorhabditis elegans*, cuticlin-1 contributes to the formation of extracellular envelopes, thereby protecting the organism from the environment [59].

It is important to note that in accordance with previous studies [11, 60, 61], the 10 dpi sera in the present study identified the protein enolase in crude ML extract. Bernal et al. [61] revealed that enolase plays a part in many processes, including fibrinolysis and degradation of the extracellular matrix, through the activation of plasminogen (a proenzyme of the serine protease plasmin). Moreover, this enzyme may contribute to tissue migration during all *T. spiralis* developmental stages [59]. Dea-Ayuela & Bolas-Fernandez [30] confirmed that enolase the immunoreactive property using a combination of 2D-immunoblot and MS. Our findings also confirm the presence of a common proteins for both *T. britovi* stages which was recognized by sera from pigs at 10 dpi and 60 dpi. One particularly well-studied group of proteins comprises the heat-shock proteins (Hsps), which are known to assist the parasite in tissue invasion and intracellular survival, as well as protect it against injury or stress conditions arising as a result of host immune response stimulation [62]. This is consistent with earlier results which identified Hsps as being a common to the adult and muscle larvae stages [10, 26, 51, 55, 63], and were recognized by sera at 15 dpi and 45 dpi [11]. The present GO analysis demonstrated that the identified Hsp proteins present oxidoreductase and structural molecule activity, and that they are located on ribosomes and take part in the translation processes, suggesting that they participate in host cellular stress and immune responses, as well as in the regulation of gene expression and parasite development [27, 64].

Our findings also indicate that the heat-shock protein beta identified in both the Ad and ML proteomes belongs to the small heat-shock proteins (sHsp), which are considered to be an important focus of research in the fight against parasitic diseases [65]. Wu et al. [66] reported that sHsp likely play a role in enhancing the survival of the *T. spiralis* muscle larvae under conditions of chemical and physical stress, as well as in the development of larvae. Wang et al. [64] suggested that recombinant Hsp70 is an immunogenic protein released by parasites and that it is exposed to the host immune system during infection.

Intermediate filament protein (IFA-1) and intermediate filament protein B were identified in both *T. britovi* proteomes. These are members of the diverse family of intermediate filaments; these are cytoskeletal components of animal cells which contribute to their mechanical strength and facilitate growth [67]. In nematodes, they allow epidermal elongation in the larvae, worm growth and muscle stability maintenance [68]. Peroxiredoxin-2 has antioxidant and oxidoreductase activity, participates in cellular oxidant detoxification processes and preserves cell redox homeostasis. It therefore plays a crucial role during the host immune response by protecting parasites from endogenous and host-derived ROS, and is possibly involved in cellular signaling [69].

The present study examined somatic extracts taken from adult worms (AW) and muscle larvae of *T. britovi*. Some of the proteins present in these somatic extracts might not be excretory-secretory (E-S) proteins, and they cannot be exposed to the host immune system and induce the specific antibody response. Hence, some of the identified proteins may have less sero-diagnostic value, or perhaps no significance at all. Nevertheless, in the process of *Trichinella* infection, the E-S antigens produced by the AW and ML are directly exposed to the immune system and elicit the production of specific anti-*Trichinella* antibodies by the host. Immunoproteomics studies have identified the early diagnostic antigens associated with the E-S proteins of *T. spiralis* AW and ML in animal or patient sera during early infection, and the recombinant 31 kDa antigen from *T. spiralis* ML E-S proteins has been proved to be valuable for early diagnosis of trichinellosis [70, 71]. Hence, further diagnostic antigens for *T. britovi* infection may be identified by future studies on the E-S antigens of AD and ML with early infection sera.

Few proteomic studies examine *T. britovi* exclusively or compare the findings with those of different *Trichinella* spp. [9], and those that have been performed focus on the characterization of mitochondrial genomes [72]. This approach results in the acquisition of a narrow range of knowledge regarding the nuclear genomic or transcriptomic data associated with this parasite, and this narrow focus presents a serious obstacle in the identification of its proteins, and the understanding of their precise function during parasite invasion. Therefore, many proteins are not represented in existing studies, and their precise function can only be assumed on the basis of indirect resemblance analysis.

## Conclusions

To our knowledge, the present study describes the first immunoproteomic identification of the antigenic proteins of adult worm and muscle larvae of *T. britovi*. The somatic extracts from adult worms and muscle larvae of *T. britovi* were specifically recognized by *T. britovi*-infected pig sera at 10 dpi and 60 dpi; a total of 70 prominent protein spots were thus identified, and these were found to contain 45 adult worm and 52 muscle larvae proteins. Adult worms and muscle larvae of *T. britovi* produce proteins (both stage-specific and common proteins) with antigenic properties, some of which have been identified in other helminths as potential diagnostic targets and vaccine candidates. The presence of common and stage-specific proteins for both investigated *T. britovi* stages was confirmed; these included heat-shock proteins, intermediate filament protein IFA-1, 32 kDa beta-galactosidase-binding lectin, peroxiredoxin-2 or 14-3-3 protein, actin-5C, paramyosin, intermediate filament protein B, calreticulin, deoxyribonuclease-2-alpha, enolase, serine protease. These proteins were related to many significant molecular functions, cellular components and biological processes of the parasite, suggesting that the somatic proteins of these two developmental stages may induce a humoral immune response, making them potential antigens for the development of diagnostic methods for *T. britovi* infection.

## Abbreviations

2-DE: Two-dimensional electrophoresis
Ad: Adult worms
dpi: Days post-infection
ELISA: Enzyme-linked immunosorbent assay
ES: Excretory-secretory
IEF: Isoelectric focusing
LC-MS/MS: Liquid chromatography-tandem mass spectrometry
ML: Muscle larvae
MW: Molecular weight
NBL: Newborn larvae
pI: Isoelectric point
PVDF: Polyvinylidene fluoride membrane
